# Digital twins in dermatology: a new era of personalized skin care

**DOI:** 10.3389/fdgth.2025.1534859

**Published:** 2025-02-10

**Authors:** Diala Haykal

**Affiliations:** Private Practice, Centre Laser Palaiseau, Palaiseau, France

**Keywords:** digital twins, dermatology, personalized medicine, skin conditions, predictive dermatology

## Introduction

The concept of digital twins, initially developed in engineering and manufacturing, is now creating a significant impact in healthcare, particularly in dermatology. A digital twin is a virtual representation of an individual's skin, designed by integrating real-time data such as imaging, genetic information, lifestyle factors, and environmental influences (1, 2). A digital twin is a common product of the Internet of Things (IoT), deep and digital phenotyping, and artificial intelligence (AI) (3). This technology promises to revolutionize dermatology by enabling highly personalized care, predictive diagnostics, and optimized treatment plans tailored to each patient's unique skin profile (2). By exploring the potential applications of digital twins, this manuscript emphasizes their transformative role in personalized treatment for skin conditions, aesthetic and anti-aging interventions, and predictive dermatology for proactive skin care. It also delves into the role of AI and big data in powering this innovation while addressing the practical challenges, ethical considerations, and future directions necessary for the successful adoption of digital twins in dermatology.

## Understanding digital twins in dermatology

A digital twin in dermatology is a dynamic, evolving virtual model that mirrors a patient's skin characteristics. It incorporates factors like skin type, hydration, elasticity, pigmentation, and environmental exposure (UV radiation, pollution) to simulate various skin conditions or responses to treatment (3). As data from sensors, wearable devices, high-resolution imaging, and electronic health records continuously update the twin, it provides real-time insights that can guide dermatological interventions (4). This evolving digital model allows dermatologists to not only diagnose and treat current skin conditions, but also predict future skin issues based on a wide range of data points (5). For instance, a patient's digital twin can track skin aging, simulating the effects of sun exposure or pollution over time, helping dermatologists tailor preventive strategies.

## Personalized dermatological treatment

While guidelines exist for treating conditions like acne and psoriasis, individual variability often necessitates iterative adjustments to achieve optimal outcomes. Digital twins can enhance the precision of these guidelines by simulating individual responses to treatments, minimizing the need for trial-and-error approaches (6). For example, in the treatment of acne, a digital twin could simulate how a patient's skin might react to topical treatments like retinoids or benzoyl peroxide, as well as oral medications such as antibiotics or hormonal therapy. By virtually experimenting with these options, dermatologists can predict which treatments will yield the best results with minimal side effects, avoiding lengthy and sometimes frustrating trial periods for the patient. Similarly, digital twins can be invaluable in managing psoriasis, where treatment efficacy can vary significantly depending on individual factors such as the patient's immune response, lifestyle, and environmental triggers (1). By simulating how different treatments, like biologics or phototherapy, would interact with the patient's unique skin and immune system, dermatologists can make data-driven decisions that optimize therapeutic outcomes (7).

## Enhancing aesthetic dermatology and anti-aging treatments

In the realm of cosmetic dermatology, digital twins provide unprecedented opportunities for personalized anti-aging treatments. Aging is influenced by numerous factors, including genetics, sun exposure, and overall health. A digital twin can incorporate all these variables to predict the patient's skin aging trajectory, helping dermatologists to recommend the most effective treatments at the right time (8). For instance, the twin can model the effects of energy-based devices such as fractional lasers or radiofrequency treatments, ensuring that the parameters used are specific to the patient's skin depth, elasticity, and collagen structure. This level of customization minimizes the risk of over- or under-treatment and maximizes the desired outcomes (8, 9).

Before the advent of digital twins, dermatologists relied heavily on their clinical experience, patient history, and standardized protocols to plan treatments. While this approach often worked well, it lacked the precision to account for individual variations in skin type, genetic predispositions, and real-time environmental factors. Treatments like injectables, resurfacing lasers, or melasma management involved a certain level of trial and error, with patients sometimes experiencing suboptimal results or unexpected side effects. Controversial procedures, such as using certain laser settings on darker skin tones or administering higher volumes of dermal fillers, presented significant challenges. Dermatologists had to balance potential benefits with risks like scarring, hyperpigmentation, or unnatural outcomes, often making decisions without the full predictive insights that digital twins now provide. The lack of highly personalized data sometimes led to patient dissatisfaction or prolonged recovery periods, as there was no way to accurately simulate how their skin would react to specific treatments before the procedure (5, 10, 11).

## Real-world examples

Although the application of digital twins in dermatology is still in its early stages, there are promising examples in related fields. For instance, digital twins are being piloted in oncology for predicting responses to cancer treatments and in cardiology for simulating patient-specific interventions. In dermatology, companies like SkinTwin**®** are exploring virtual skin modeling, while certain academic groups are integrating digital twins with AI for melanoma research (12). Additionally, companies such as Viz.ai® and Butterfly Network® are leveraging digital twin principles in diagnostics and imaging, with potential applications in dermatology to map skin lesions and forecast disease progression (13, 14). Highlighting these efforts underlines the potential for translating this technology into routine dermatological practice.

## Specific examples of utilization and comparisons with gold standards

Digital twin technology has shown promising applications in dermatology, bridging the gap between theoretical predictions and practical outcomes. For instance, digital twins have been utilized to simulate the response of acne-prone skin to various treatments, including topical retinoids, hormonal therapies, and laser treatments (15, 16). This allows for tailoring the intervention to the patient's unique skin profile, reducing the frustration of trial-and-error treatment methods (17, 18). Similarly, in managing hyperpigmentation, digital twins can forecast patient outcomes with energy-based devices, such as picosecond lasers, by modeling the interaction between laser parameters and the patient's melanin concentration (5). Compared to current gold-standard treatments like fractional CO2 lasers or oral isotretinoin, digital twins provide a data-driven approach that minimizes side effects and optimizes results, offering significant advantages in patient satisfaction and treatment efficiency.

## Predictive dermatology: proactive skin care

One of the most transformative applications of digital twins in dermatology is their potential for predictive care. By integrating genetic data, environmental exposure, and patient lifestyle, digital twins can forecast the likelihood of future skin issues and help dermatologists develop proactive care plans (19). For example, in melanoma monitoring, digital twins can continuously analyze skin lesions for subtle changes indicative of malignancy, potentially detecting skin cancer at its earliest stages. This ongoing surveillance allows for earlier interventions, which are often less invasive and more effective. Patients with a genetic predisposition to skin cancer could benefit further from digital twin simulations predicting cumulative sun exposure's effects, enabling dermatologists to design tailored prevention strategies involving sun protection measures, regular screenings, and specific skin care routines (3, 20). In chronic conditions like rosacea or hyperpigmentation, digital twins can analyze genetic markers, environmental factors, and patient history to predict flare-ups or pigmentation issues (5). This predictive capability allows dermatologists to prescribe preventive treatments or recommend lifestyle adjustments before conditions worsen. Such an approach reduces the need for frequent clinical visits, enhances patient outcomes, and fosters greater patient satisfaction by enabling proactive care. Despite these advantages, digital twin adoption in dermatology has been comparatively slower than in fields like oncology, which faces similar challenges such as integrating large datasets and addressing patient-specific variability (21, 22). This disparity can be attributed to several factors. Dermatology has traditionally relied on visual and tactile assessments, which, while effective, may not fully leverage the computational precision offered by advanced technologies (23). Additionally, many dermatological conditions, though impactful, are not life-threatening, leading to a slower pace of clinical validation compared to oncology, where timely innovation is often critical.

Another barrier is the lack of digital infrastructure in dermatology clinics. While oncology departments in leading hospitals may possess systems for genomic sequencing and real-time monitoring, dermatology clinics often rely on less advanced tools, making the integration of digital twin technology challenging, particularly in resource-constrained settings (3). Furthermore, there is often a lack of standardized protocols for creating and maintaining digital twins specific to dermatological needs, which can deter widespread implementation. To overcome these challenges, it is critical to emphasize the practical benefits of digital twins in dermatology. For example, demonstrating their ability to predict flare-ups in conditions such as psoriasis or rosacea through pilot programs can showcase tangible benefits to both clinicians and patients (24). These programs can engage stakeholders and build trust in the technology. Highlighting the cost-effectiveness of digital twins, such as minimizing trial-and-error in treatment plans and reducing overall patient care costs, can further encourage adoption (9). Lastly, fostering collaborations between technology developers, dermatologists, and regulatory bodies is essential to establish accessible, standardized, and practical protocols for integrating digital twin technology into everyday clinical use (10).

## Practical adoption steps

The successful adoption of digital twin technology in dermatology begins with integrating AI-powered imaging devices, such as dermoscopy systems, high-resolution imaging tools, and wearable biosensors. Equipped with deep learning algorithms, these devices continuously analyze skin parameters like pigmentation, hydration, elasticity, and lesion morphology (1, 2, 4). The resulting data feeds into digital twin models, enabling real-time updates and predictive insights for personalized treatment plans. For example, these tools can detect subtle changes in skin pigmentation, providing early warnings for conditions like melasma or hyperpigmentation and allowing for timely intervention (4, 5, 25). To facilitate implementation, a comprehensive training program is crucial. This should include theoretical modules on AI and digital twin principles, practical workshops on device operation and data interpretation, and simulation exercises in mock clinical scenarios, such as managing acne or psoriasis (3). These initiatives provide dermatologists with hands-on experience, building their confidence to integrate digital twin technology into routine practice and make data-driven decisions that optimize patient outcomes. By combining advanced imaging technologies with robust training, dermatology practices can overcome adoption barriers and unlock the transformative potential of digital twin technology (7, 8, 26).

## AI and big data: empowering digital twins in dermatology

AI algorithms are capable of processing vast amounts of data, from imaging, genetic tests, and patient histories, to identify patterns that are impossible for humans to detect. The high analytical capacity of AI founded in deep learning can empower aesthetic dermatological tools to attain precise prediction. This predictive power allows digital twins to provide tailored, data-driven insights (8). In melanoma detection, for example, AI can analyze thousands of skin lesion images, using deep learning algorithms to differentiate between benign moles and malignant melanomas (17). This data feeds into the digital twin, which can then alert both patients and dermatologists to any concerning changes, improving early detection rates (7). AI also assists in refining treatment plans, optimizing parameters for laser treatments, or predicting the outcome of combination therapies in complex conditions like acne or psoriasis (27).

## Challenges and ethical considerations

**Chart 1:**
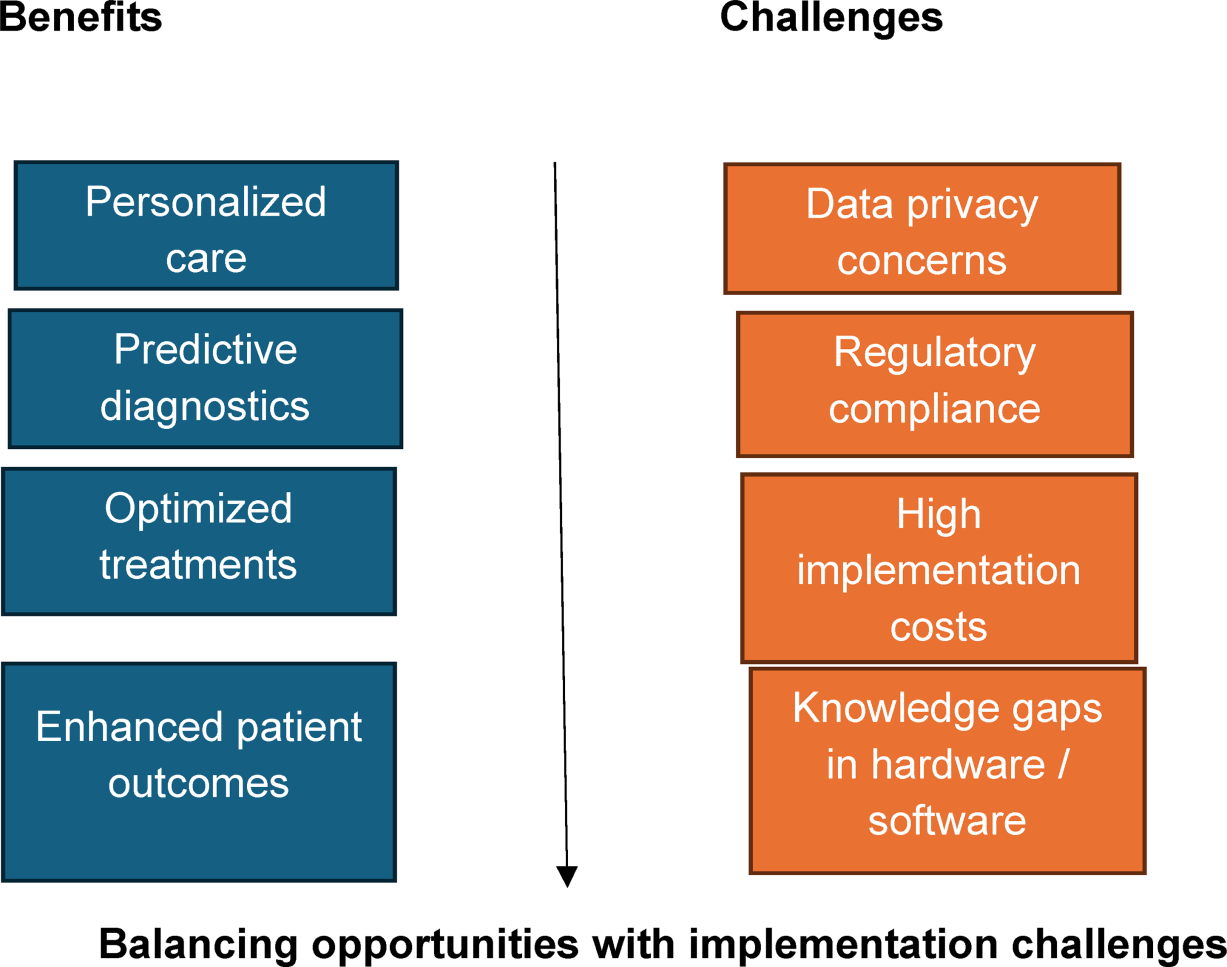
Benefits and challenges of digital twins in dermatology.

While the potential of digital twins in dermatology is immense, several challenges must be addressed (28). First, data privacy is a critical issue. The creation and maintenance of digital twins requires continuous collection of sensitive personal health information, including images and genetic data. Ensuring the security of this data, especially in an era of increasing cyber threats, is paramount. Another challenge is the integration of digital twins into clinical practice. Dermatology clinics, particularly in resource-limited settings, may struggle to implement the infrastructure necessary for real-time data collection and AI analysis. The technology requires significant investment in hardware, software, and workforce training, which may not be feasible for all healthcare providers. Moreover, the regulatory landscape surrounding digital twins is still in its infancy. To ensure the widespread adoption of this technology, regulatory bodies must establish clear guidelines regarding data handling, clinical validation, and ethical considerations (29). For example, questions of liability may arise if a treatment recommended by a digital twin proves ineffective or harmful, raising concerns about how to balance machine and human decision-making in medical practice. A major barrier to integrating digital twins into dermatology is the gap in knowledge about the necessary hardware and software. Standardization efforts should include identifying minimal requirements, such as AI-integrated imaging systems, secure cloud storage for patient data, and expertise in data science. Policymakers must also provide guidelines for clinics to adopt digital twins safely and cost-effectively. Additionally, the regulatory landscape must evolve to address liability concerns and ensure clear ethical frameworks are in place. Addressing these challenges through collaborative efforts between healthcare providers, policymakers, and technology developers is essential to unlock the full potential of digital twins in dermatology and ensure equitable access for all patients. Chart 1 illustrates some benefits and challenges of digital twins in dermatology.

## The future of digital twins in dermatology

Despite these challenges, the future of digital twins in dermatology looks promising. As AI algorithms become more sophisticated, and as more data is integrated into digital twin models, the accuracy and predictive power of this technology will continue to improve. This will likely lead to more widespread adoption of digital twins, both for personalized skin care and for population-level health management (30). Furthermore, the potential for digital twins to be used in clinical trials for dermatological treatments could revolutionize the speed and efficiency of drug development (26). By simulating how large populations of digital twins respond to new treatments, pharmaceutical companies could accelerate the discovery process, reducing the need for extensive human trials. Lastly, digital twins are currently being used in construction and Silicon Valley type of industries; the field of dermatology seems to have lagged in adopting such a turning point technology as digital twin. Therefore, the process of developing digital twin creation protocols suitable for the field of dermatology requires prompt acceleration.

## Conclusion

Digital twins indicate a groundbreaking promise of advancement in dermatology, offering unprecedented opportunities for personalized skin care and predictive treatments. From optimizing cosmetic procedures to proactively managing chronic skin conditions, digital twins promise to transform how dermatologists approach patient care. However, as this technology continues to evolve, it will be crucial to address data privacy concerns, regulatory challenges, and accessibility issues to ensure that digital twins can benefit patients across all demographics. By harnessing the power of AI and big data, digital twins offer a glimpse into the future of personalized dermatology, where every patient receives precisely the care they need, tailored to their unique skin biology and environmental factors. As the technology continues to mature, digital twins have the potential to redefine standards of care in dermatology, improving outcomes and enhancing the patient experience.
